# Down Syndrome: Neuropsychological Phenotype across the Lifespan

**DOI:** 10.3390/brainsci11111380

**Published:** 2021-10-21

**Authors:** Margaret Pulsifer

**Affiliations:** Department of Psychiatry, Massachusetts General Hospital, Harvard Medical School, Boston, MA 02114, USA; mpulsifer@mgh.harvard.edu

Down syndrome (DS), caused by triplication of chromosome 21, is the most common genetic cause of intellectual disability (ID), with an estimated incidence of one in 700 live births. Individuals with DS commonly exhibit unique neuropsychological profiles that emerge during specific developmental stages across the lifespan, often characterized by early developmental delay, cognitive strengths and weaknesses, behavior and mental health issues and age-related cognitive decline frequently resulting in early onset Alzheimer’s disease. These profiles are unique compared to other individuals with ID and reflect the genetic mechanisms and neuroanatomic features underlying the distinct neuropsychological phenotype associated with DS. Understanding this neuropsychological phenotype across the lifespan and the associated clinical, educational and treatment needs is particularly important because of recent increases in life expectancy due to improved health care, advocacy, services and societal changes.

Individuals with Down syndrome often face unique developmental, cognitive and behavioral challenges at various stages of development across the lifespan. This Special Issue contains original research and comprehensive reviews that address a broad range of topics related to DS, including early developmental trajectories, social challenges and autism spectrum disorder, acute developmental regression, psychiatric issues and treatment, assessment and diagnosis, early onset dementia and medical co-morbidities.

Communication skills in infants with DS are often delayed compared to typically developing infants, but little is known about what factors are associated with this developmental delay. In a unique study, Pejovic et al. [1] compared 5–7-month-old infants with DS to typically developing infants in terms of visual attention and audiovisual speech processing and found that the infants with DS were slower to orient visual attention and less attentive to social/speech cues. Those differences might be related to delays in subsequent communication development. The findings could help direct more effective interventions, such as providing more time for infants with DS to attend to communicative cues and promoting attention to cues by emphasizing face-to-face communication.

Examining speech fluency, Naess et al. [2] found significantly greater difficulties with fluency in a group of children with DS with an average age of 6 years compared to typically developing children with a similar level of nonverbal cognitive functioning. Poorer performance in speech fluency was associated in the study with lower language skills. The findings highlight the need for interventions that target both speech fluency and language development in children with DS.

Another study by Naess et al. [3] examined reading skills in a national cohort of school aged children with DS in Norway, age 6 to 8 years. Children with DS often experience reading difficulties; the study’s goal was to identify factors that were associated with better decoding skills by 3rd grade. The most important factors, after controlling for nonverbal cognitive abilities, were vocabulary and letter knowledge. Those findings should help guide early educational interventions.

Autism spectrum disorder (ASD) is more commonly diagnosed in individuals with DS than in the general population. However, little is known about the unique presentation of ASD symptoms in DS. In a study examining the prevalence and presentation of ASD in a large cohort of DS individuals, age 6 to 23 years, Dimachkie Nunnally et al. [4] found that 37% of the cohort were classified as ASD on a semi-structured standardized assessment. That ASD group was marked by greater challenges with social communication and complex expressive language than those without ASD.

Children with DS, with and without ASD, experience social challenges which may result in social isolation and may impact mental health outcomes. Therefore, accurate measures of social cognition and social behavior are particularly important for children and adolescents with DS. Schworer et al. [5] addressed this need by examining the psychometric properties of several measures in a group of individuals with DS, age 6 to 17 years. One measure, the Social Responsiveness Scale-2, was particularly promising for this population.

Levels of daily living skills and independence vary in individuals with DS across the lifespan. In a large cohort of pediatric and adult individuals with DS, Krell et al. [6] found that 87% communicated verbally and fewer (17%) could use written communication. The life skills reported as most important to both adolescents and adults included learning about healthy food, preparing meals and describing symptoms to a doctor. The authors recommended that life skills should be routinely assessed during a medical appointment to support greater independence for individuals with DS.

Between teenage years and the mid to late 20 s, some individuals with DS experience an acute regression in skills and behavior. There is limited understanding of the cause of this unexplained condition and its diagnosis, treatment and prognosis. Two articles in this Special Issue examine regression in DS; one, by Walpert et al. [7], presents a review of 13 articles on early regression in DS and describes symptom presentation, potential trigger events and prognosis. The other, by Handen et al. [8], uses biomarker risk measures for Alzheimer’s disease (AD) to examine the possibility that early regression in adults with DS might lead to increased risk of AD in later life. The findings have important implications for recognition of the condition and highlight the need for further research to focus on prevention and treatment.

Depression is common in individuals with DS, but treatment with serotonin reuptake inhibitors (SSRIs) has not been closely examined in this population. In a retrospective chart review, Thom et al. [9] evaluated the effectiveness, tolerability and safety of SSRIs for depression in a cohort of adults with DS. The majority of patients in the study responded to a 12-week course of SSRI treatment, with some tolerating long-term use.

Adults with DS have an especially high risk for developing early onset AD. Accurate identification and assessment of cognitive decline in this population are essential for diagnosis and planning. Two articles in this Special Issue, by Hom et al. and Harp et al., examine the effectiveness of multiple measures of cognitive and behavioral functioning and the domains assessed in identifying dementia classification. The research by Hom et al. [10] shows that cognitive functioning can be characterized at the cognitive domain level, with language, executive functioning and memory being candidates for most impacted domains. Building on this concept, Harp et al. [11] developed an abbreviated test battery to identify individuals with DS at risk for AD.

AD and hypothyroidism are equally prevalent in adults with DS, but the relationship between the age of onset of hypothyroidism and that of AD has not been established. In a study that is the first to explore this relationship, Lai et al. [12] suggest that early onset hypothyroidism in DS is significantly associated with an early onset age of AD, independent of several co-occurring conditions of interest, including APOE Ɛ4 allele status. The authors recommend early testing of thyroid functions in adults with DS and emphasize the need for future studies to determine how hypothyroidism affects AD risk and onset.

Thanks are due to all of the authors contributing to this Special Issue. Their work represents meaningful advances in the understanding of Down syndrome throughout the lifespan, with benefits both for research and for clinicians working to improve the well-being of individuals with Down syndrome.
